# Protective Effects of Hydrolyzed Nucleoproteins from Salmon Milt against Ethanol-Induced Liver Injury in Rats

**DOI:** 10.3390/md14120232

**Published:** 2016-12-19

**Authors:** Akiko Kojima-Yuasa, Mayu Goto, Eri Yoshikawa, Yuri Morita, Hirotaka Sekiguchi, Keita Sutoh, Koji Usumi, Isao Matsui-Yuasa

**Affiliations:** 1 Department of Food and Human Health Sciences, Graduate School of Human Life Science, Osaka City University, Osaka 558-8585, Japan; v-oooogue@ezweb.ne.jp (M.G.); eri92011@gmail.com (E.Y.); morimaru.y@gmail.com (Y.M.); yuasa-i@hotmail.co.jp (I.M.-Y.); 2 Life Science Institute Co., Ltd., Tokyo 103-0012, Japan; sekiguchi@life-science.co.jp (H.S.); sutoh@life-science.co.jp (K.S.); usumi@life-science.co.jp (K.U.)

**Keywords:** DNA-rich nucleic acid prepared from salmon milt (DNSM), in vivo ethanol-carbon tetrachloride cirrhosis model, plasma aminotransferases (AST and ALT), collagen accumulation, CYP2E1 activity, alcohol-induced liver injury, rats

## Abstract

Dietary nucleotides play a role in maintaining the immune responses of both animals and humans. Oral administration of nucleic acids from salmon milt have physiological functions in the cellular metabolism, proliferation, differentiation, and apoptosis of human small intestinal epithelial cells. In this study, we examined the effects of DNA-rich nucleic acids prepared from salmon milt (DNSM) on the development of liver fibrosis in an in vivo ethanol-carbon tetrachloride cirrhosis model. Plasma aspartate transaminase and alanine transaminase were significantly less active in the DNSM-treated group than in the ethanol plus carbon tetrachloride (CCl_4_)-treated group. Collagen accumulation in the liver and hepatic necrosis were observed histologically in ethanol plus CCl_4_-treated rats; however, DNSM-treatment fully protected rats against ethanol plus CCl_4_-induced liver fibrosis and necrosis. Furthermore, we examined whether DNSM had a preventive effect against alcohol-induced liver injury by regulating the cytochrome p450 2E1 (CYP2E1)-mediated oxidative stress pathway in an in vivo model. In this model, CYP2E1 activity in ethanol plus CCl_4_-treated rats increased significantly, but DNSM-treatment suppressed the enzyme’s activity and reduced intracellular thiobarbituric acid reactive substances (TBARS) levels. Furthermore, the hepatocytes treated with 100 mM ethanol induced an increase in cell death and were not restored to the control levels when treated with DNSM, suggesting that digestive products of DNSM are effective for the prevention of alcohol-induced liver injury. Deoxyadenosine suppressed the ethanol-induced increase in cell death and increased the activity of alcohol dehydrogenase. These results suggest that DNSM treatment represents a novel tool for the prevention of alcohol-induced liver injury.

## 1. Introduction

Alcoholic liver disease is a pathological process characterized by progressive liver damage that leads to steatosis, steatohepatitis, fibrosis, and ultimately cirrhosis, which may further progress to hepatocellular cancer [1,2,3]. Oxidative stress plays an important role in this process [4,5]. Alcohol-induced oxidative stress associated with ethanol metabolism in the liver plays a major role in ethanol-induced liver injury. Alcohol dehydrogenase is the major enzyme responsible for oxidizing ethanol to aldehyde in alcohol metabolism. Heavy consumption of ethanol induces cytochrome p450 2E1 (CYP2E1) activity in hepatocytes. This enzyme complements the activity of a constitutively expressed alcohol dehydrogenase in oxidizing ethanol to acetaldehyde [6]. However, CYP2E1 generates reactive oxygen species (ROS) quite efficiently, which appears to play a major role in ethanol-induced liver injury [7,8,9,10]. Therefore, possible strategies for preventing the production of these ROS may be effective in attempts to minimize the hepatotoxicity of ethanol in humans.

Animal models of liver fibrosis are important for understanding the underlying mechanisms of the treatments used to combat this disease. Currently, two models have been developed for administering alcohol to animals: the Lieber-De Carli liquid diet [11] and the Tsukamoto-French gastric model [12]. In the Lieber-De Carli liquid diet, ethanol replaces the carbohydrates of a normal diet. Tsukamoto and French developed an in vivo animal model in which enteral ethanol is continuously administered to the animal via intragastric infusion. However, neither the Lieber De Caarli nor the Tsukamoto/French feeding protocol results in cirrhosis in rats. Tipoe et al. reported another model for administering dietary alcohol and fish oil (30% of calories) and showed that an increase in profibrogenic mediators was not associated with the presence of histological evidence of fibrosis [13]. Contrastingly, Siegers et al. developed a model for the administration of low-dose carbon tetrachloride (CCl_4_) and a 5% ethanol solution that produced histological changes in rats similar to those found in human alcoholic cirrhosis within four weeks [14]. We have also shown that hepatic histological changes occurred within four weeks of the administration of low-dose CCl_4_ and a 5% ethanol solution [15,16].

Salmon milts contain mainly nucleic acids, protamine, and polyamine. These components play an important role in the diet. Dietary nucleic acids are particularly important for the development and growth of tissues. Nucleic acids are partly degraded by nucleases in the intestine and absorbed as nucleosides and nucleobases. Dietary nucleotides play a role in maintenance of immune responses in both animals and humans [17,18,19]. Several researchers have also reported that orally administered nucleic acids from salmon milt play a role in physiological functions such as cellular metabolism, proliferation, differentiation, and apoptosis in human small intestinal epithelial cells [17,20,21]. Additionally, Sakai et al. have reported that dietary ribonucleic acid (RNA) suppresses inflammation in adipose tissue and improves glucose intolerance in mice fed a high-fat diet [22].

In this study, we examined the effect of DNA-rich nucleic acids prepared from salmon milt (DNSM) on the development of liver fibrosis in an in vivo ethanol-CCl_4_-induced cirrhosis rat model and in an in vitro alcohol-injury hepatocytes model, and we found that DNSM protected hepatocytes against ethanol induced liver injury.

## 2. Results

**Figure 1:**
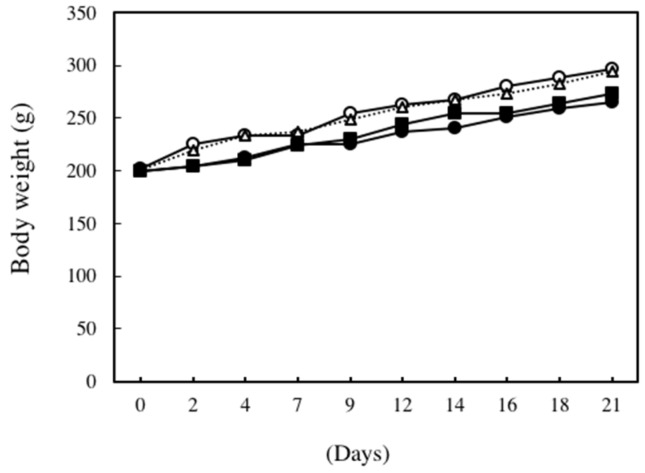
Changes in body weight. ○: Control diet, ●: Control diet with 5% ethanol plus CCl_4_, △: Control diet with CCl_4_, ■: 0.12% DNSM diet with 5% ethanol plus CCl_4_.

Figure 1 shows changes in body weight during the experimental period. The body weights of ethanol plus CCl_4_-treated rats and 0.12% DNSM diet- and ethanol plus CCl_4_-treated rats tended to be lower than those rats fed the control diet or CCl_4_ alone. However, these differences were not statistically significant.

**Figure 2:**
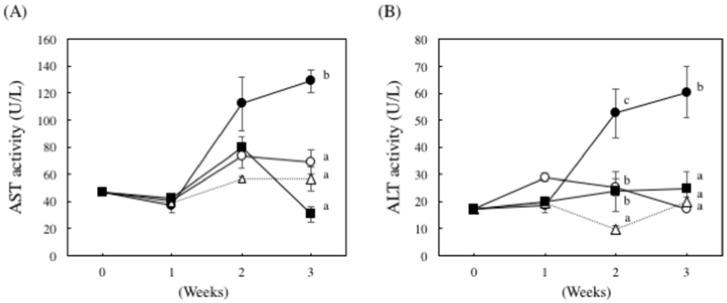
The effect of DNSM on serum AST and ALT activity in ethanol plus CCl_4_-treated rats. Effect of DNSM on (**A**) serum AST activity; and (**B**) serum ALT activity. Data are presented as the mean ± S.E. of the activity of five rats. Values without a common letter are significantly different (*p* < 0.01). ○: Control diet, ●: Control diet with 5% ethanol plus CCl_4_, △: Control diet with CCl_4_, ■: 0.12% DNSM diet with 5% ethanol plus CCl_4_.

We examined the effect of nucleoprotein treatment on plasma aspartate aminotransferase (AST) and alanine aminotransferase (ALT) activities. As shown in Figure 2, in the ethanol plus CCl_4_ (0.1 mL/kg of body weight)-treated group, plasma AST and ALT activities increased by 1.8- and 3.5-fold, respectively, compared to the control group. However, these same enzymes were significantly less active in the DNSM-treated group compared to the ethanol plus CCl_4_-treated group.

**Figure 3:**
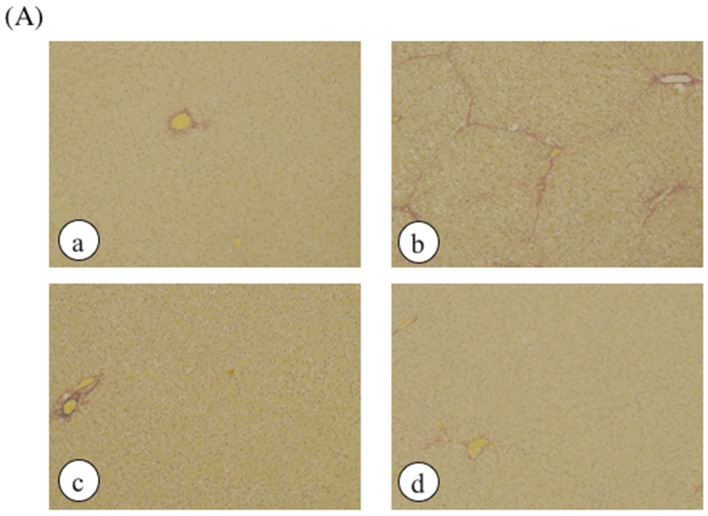

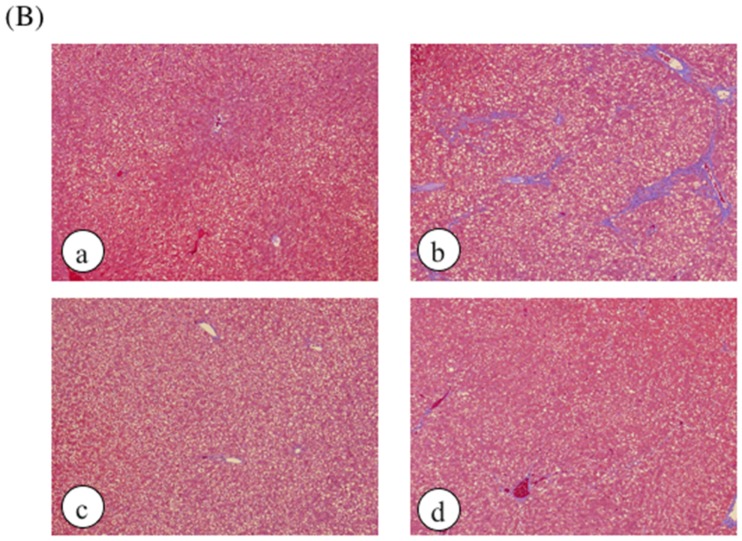
The effect of DNSM on the changes in liver morphology. Liver sections were processed for (**A**) EVG staining; and (**B**) Masson’s trichrome staining. (**a**) Control diet; (**b**) Control diet with 5% ethanol plus CCl_4_; (**c**) Control diet with CCl_4_; (**d**) 0.12% DNSM diet with 5% ethanol plus CCl_4_.

Histological analysis was performed by hematoxylin and eosin staining as well as elastic van Gieson (EVG) staining and Mason’s trichrome staining to assess liver damage (Figure 3). No histological abnormalities were observed in the control rats or CCl_4_-treated rats, but collagen accumulation in the liver and hepatic necrosis were observed in ethanol plus CCl_4_-treated rats. However, treatment with DNSM fully protected the rats from liver fibrosis and necrosis induced by ethanol plus CCl_4_.

**Figure 4:**
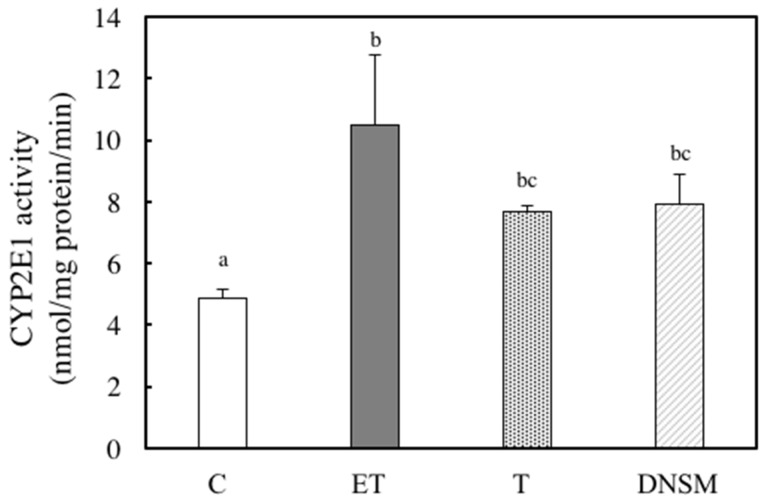
The effect of DNSM on CYP2E1 activity in the livers of ethanol plus CCl_4_-treated rats. CYP2E1 activity was determined using the ρ-nitrophenol (PNP) assay, as described in the Materials and Methods. (C) Control diet; (ET) Control diet with 5% ethanol plus CCl_4_; (T) Control diet with CCl_4_; (DNSM) 0.12% DNSM diet with 5% ethanol plus CCl_4_. Data are presented as the mean ± S.E. of five animals. Values without a common letter are significantly different (*p* < 0.01).

**Figure 5:**
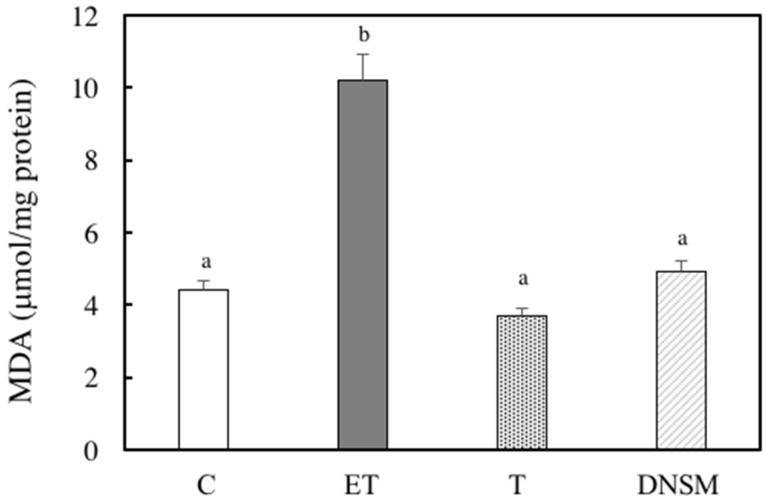
The effect of DNSM on lipid peroxidation in the liver. The measurement of lipid peroxidation using a colorimetric reaction with thiobarbitric acid (TBA) was carried out according to the method described by Ohkawa. The measured lipid peroxidation was expressed as malondialdehyde (MDA). (C) Control diet; (ET) Control diet with 5% ethanol plus CCl_4_; (T) Control diet with CCl_4_; (DNSM) 0.12% DNSM diet with 5% ethanol plus CCl_4_. Each bar is the mean ± S.E. of five animals. Values without a common letter are significantly different (*p* < 0.01).

Ethanol-induced oxidative stress from CYP2E1 appears to play a major role in ethanol-induced liver injury [7,8,9,10]. We have previously demonstrated that Yerba mate extract suppressed the CTP2E1 activity induced by ethanol in both in vitro and in vivo models [16]. Therefore, we examined whether DNSM treatment could also have a preventive effect against alcohol-induced liver injury by regulating the CYP2E1 enzyme in an in vivo model. In ethanol plus CCl_4_-treated rats, CYP2E1 activity increased 2.1-fold compared to the control group. However, DNSM-treatments suppressed CYP2E1 activity (Figure 4). We examined the effect of DNSM on the increase in intracellular lipid peroxidation using the thiobarbituric acid reactive substances (TBARS) assay. In ethanol plus CCl4-treated rats, hepatic TBARS levels were significantly increased. However, DNSM-treatment maintained the intracellular TBARS levels at the lower levels of the control rats (Figure 5).

Furthermore, we measured the weights of various organs after the experimental period. As shown in Table 1, there were no differences among the weights of liver, kidney, and spleen in rats of the four groups. However, the masses of epididymal fat and visceral fat of ethanol plus CCl_4_-treated rats and DNSM diet- and ethanol plus CCl_4_-treated rats were lower than those rats fed the control diet or CCl_4_ alone. These results suggest that loss of body weight in ethanol plus CCl_4_-treated rats and DNSM diet- and ethanol plus CCl_4_-treated rats may depend on the loss of fat and the loss may be induced by ethanol ingestion.

**Figure 6:**
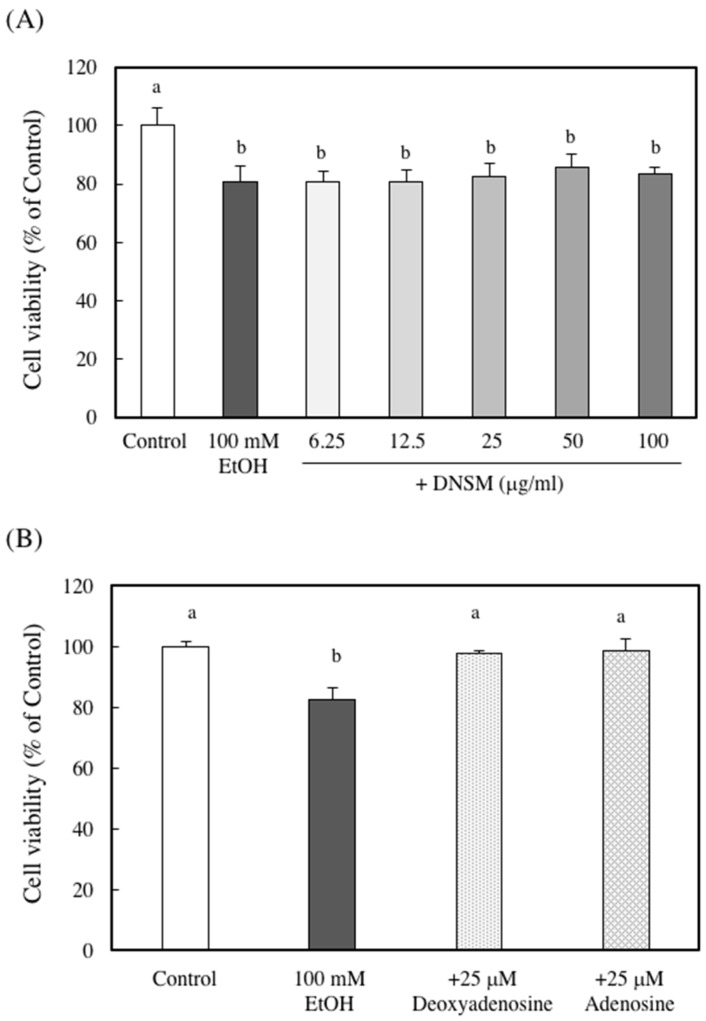
The effect of DNSM on ethanol-treated hepatocyte cell viability. Hepatocytes were incubated with 100 mM ethanol with or without (**A**) various DNSM concentrations; and (**B**) deoxyadenosine or adenosine for 24 h. Cell viability was measured by the Neutral Red assay, as described in the Materials and Methods section. Data are presented as the mean ± S.E. of three experiments. Values without a common letter are significantly different (*p* < 0.01).

**Figure 7:**
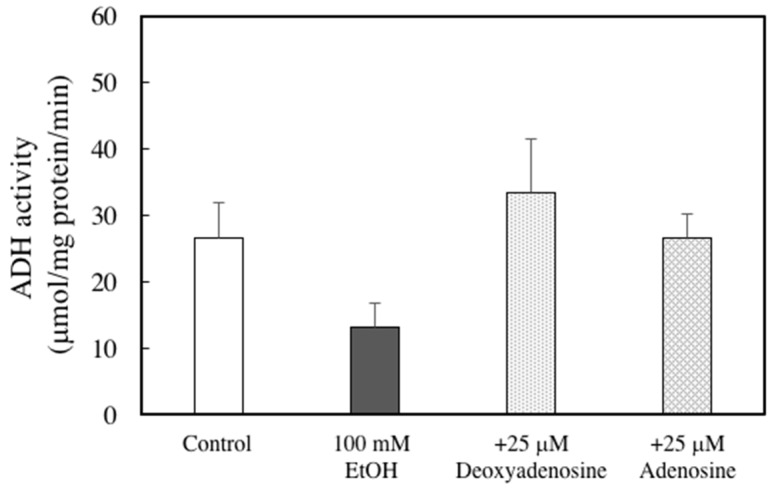
Effects of adenosine or deoxyadenosine on ADH activity in ethanol-treated hepatocytes. Hepatocytes were incubated for 4 h with 100 mM ethanol, with or without 25 μM deoxyadenosine or adenosine. ADH activity analysis was performed as described in the Materials and Methods section. Data are presented as the mean ± S.E.

To elucidate whether the protective effect of DNSM is dependent on digestion, we measured the effect of DNSM treatment in an in vitro alcohol-induced injury model in hepatocytes. We previously demonstrated that a treatment of 100 mM ethanol for 24 h significantly decreased cell viability of hepatocytes compared with control cells [15]. Here, we measured the cell viability of hepatocytes treated with 100 mM ethanol with or without various concentrations of DNSM. As shown in Figure 6A, DNSM did not prevent cell death. These results suggest that digestive products of DNSM are effective for the prevention of alcohol-induced liver injury. Therefore, the following experiments were carried out with the presence of deoxyadenosine. Adenosine which is the digestive product of RNA also was measured. Treatments of deoxyadenosine and adenosine prevented cell death in the hepatocytes treated with 100 mM ethanol (Figure 6B). Furthermore, the effect of deoxyadenosine and adenosine on the activity of alcohol dehydrogenase (ADH)—a main pathway of alcohol metabolism—was examined in the cells incubated for 4 h with 100 mM ethanol. The treatment of deoxyadenosine and adenosine increased the activities of ADH compared with the cells treated with 100 mM ethanol (Figure 7).

## 3. Discussion

This study has shown that DNSM protects against ethanol-induced liver injury. Here, we demonstrated that the liver damage biomarkers, ALT and AST, were increased in a rat model given ethanol plus CCl_4_ to induce liver damage, but this increase was reduced by nucleic acid supplementation. Furthermore, the DNSM treatment did not affect cell viability in ethanol-treated hepatocytes, suggesting the digestive products, which are created by the degradation of DNSM by nucleases in the intestine, are effective against alcohol-induced liver injury.

Alcohol-induced liver injury is induced by heavy drinking and is accompanied by the degeneration or necrosis of hepatocytes, which disrupts normal liver function via oxidative stress. The CYP2E1 enzyme is one of the major producers of ethanol-induced ROS. Therefore, decreasing or inhibiting CYP2E1 activity may be a feasible strategy for minimizing the hepatotoxicity of ethanol. Recently, we demonstrated that *Ecklonia cava* polyphenol-treatment maintained CYP2E1 activity in ethanol-treated hepatocytes below that of control cells [23]. We also reported that treatment with an extract of Yerba Mate tea suppressed ethanol-induced increases in CYP2E1 activity to the level of the control cells in an in vitro, alcohol-induced hepatocyte model and an in vivo ethanol plus CCl_4_-induced liver-injury model [16].

In the present study, we have shown that DNSM treatment suppressed ethanol-induced increases in CYP2E1 activity to the activity levels observed in the control rats and that the treatment of deoxyadenosine increased the ADH activity compared with the cells treated with ethanol in an in vitro alcohol injury hepatocyte model.

There are several reports that adenosine and deoxyadenosine has a protective effect against various diseases. Modis et al. reported that adenosine and its metabolite, inosine, exerted cytoprotective effects in an in vitro model of liver ischemia-reperfusion injury [24]. Hasemi et al. have shown that adenosine and deoxyadenosine induces apoptosis in human breast cancer cells via the activation of the mitochondria/intrinsic apoptotic pathway [25]. Furthermore, Lee reported that adenosine protected Sprague-Dawley rats from a high-fat diet and repeated acute restraint stress-induced intestinal inflammation and altered expression of nutrient transporters [26].

In the present study, the precise mechanism of the DNSM protection against ethanol-induced liver injuries in rats is not clear. However, there are two possible explanations for this protection. The first is via the cyclic AMP (cAMP)/protein kinase A (PKA) pathway. There are some reports that the cAMP/PKA signaling pathway regulates the activities of alcohol dehydrogenase and CYP2E1 in ethanol metabolism [27,28]. The administration of theophylline to rats, which inhibits cAMP phosphodiesterase and thus increases endogenous cAMP levels, or the addition of dibutyryl cAMP to hepatocyte cultures, both increased ADH activity [27]. Contrastingly, cAMP-dependent phosphorylation of CYP2E1 lead to a reduction in CYP2E1 activity [28]. We have shown that the treatment of *Ecklonia cava* polyphenol with ethanol increased the activity of alcohol dehydrogenase and inhibits CYP2E1 activity [23]. The changes in CYP2E1 and alcohol dehydrogenase activity were suppressed by treatment with H89, an inhibitor of PKA, suggesting that *Eclonia cava* polyphenol has a protective effect against ethanol-induced liver injury in a cAMP-dependent manner.

Charest et al. showed that adenosine and AMP increased cAMP concentration by interacting with the adenosine receptor [29,30]. These results suggest that DNSM was digested by nucleases in the intestine and absorbed as nucleosides and nucleobases. Adenosine and AMP subsequently bind to the adenosine receptor, which activates adenylate cyclase.

Another possibility is the involvement of the adenosine monophosphate-activated protein kinase (AMPK) signaling pathway. AMPK is a sensor that regulates cellular metabolism and oxidative stress [31,32]. Chronic alcohol consumption results in inhibition of the hepatic AMPK signaling pathway by ethanol, which leads to steatosis [33]. Fat accumulation in hepatocytes leads to the development of fatty liver. With continued alcohol consumption, fatty liver may progress to hepatitis and cirrhosis. Therefore, it is important to enhance AMPK pathway signaling to protect the liver against diseases induced by ethanol. Shin et al. have reported that β-lapachone, a naturally occurring quinone, activated the AMPK pathway in ethanol-fed rats [34]. Wang et al. also indicated that oligomeric proanthocyanidines, a class of flavonoid compounds, alleviated liver steatosis and damage through AMPK activation against alcohol-induced liver steatosis and injury [35]. On the other hand, Stenesen et al. reported that dietary adenine controlled adult lifespan via adenosine nucleotide biosynthesis and AMPK activation [36]. Dietary adenine feeding increases the ratio of AMP:ATP and ADP:ATP and activates AMPK. These results suggest that nucleoproteins activate the AMPK signaling pathway in the liver.

It is important to know the precise mechanism of the DNSM protection against ethanol-induced liver injuries in rats. Especially, the involvement of cAMP/PKA pathway or AMPK pathway in an in vivo ethanol-carbon tetrachloride cirrhosis model need to be elucidated further.

## 4. Materials and Methods

### 4.1. Materials

DNA-rich nucleic acids from salmon milt (DNSM) were water-solubilized using nuclease and protease. The nucleotide and amino acid composition of the DNSM is shown in Table 2. DNSM were provided by Fordays Co., Ltd. (Tokyo, Japan) and L•S Corporation. Williams’ Medium E and β-nicotinamide adenine dinucleotide hydrate were obtained from Sigma-Aldrich Co. (St. Louis, MO, USA). Fetal bovine serum (FBS) was purchased from Nichirei Biosciences, Inc. (Tokyo, Japan). The other chemicals used in this study were special-grade commercial products purchased from WAKO Pure Chemical Co., Ltd. (Osaka, Japan).

### 4.2. Animals

Male Wistar rats were purchased from Japan SLC Inc. (Shizuoka, Japan). The rats were housed at a constant temperature and were allowed free access to water and standard rat chow (LaboMR stock, Japan SLC, Inc. Shizuoka, Japan). All animal experiments followed our institution’s criteria for the care and use of laboratory animals in research, which meet the guidelines for animal experimentation at Osaka City University.

### 4.3. Animal Experiments

Male Wistar rats weighing 180–210 g were fed a standard laboratory diet and water ad libitum until three days prior to the experiment. The rats were then fed a control diet for three days and divided into four groups. Group 1 was the control; Group 2 was treated with ethanol and CCl_4_; Group 3 was treated with CCl_4_ alone; Group 4 was treated with ethanol, CCl_4_, and 0.12% DNSM. The composition of each diet is presented in Table 3. CCl_4_ (0.1 mL/kg of body weight diluted with olive oil to 25%) was administered by intraperitoneal injection twice a week (on Mondays and Thursdays), and 5% ethanol was administered in the drinking water ad libitum. The rats were euthanized after three weeks.

### 4.4. Histological Analysis

Liver samples were collected from each rat, fixed in 10% buffered formalin fixative, and then dehydrated in a graded alcohol series. Following xylene treatment, the specimens were embedded in paraffin blocks and cut into 5-μm sections. Consecutive sections were stained with EVG and Masson’s trichrome staining. The pathologist was blinded to the rats’ group assignments.

### 4.5. Liver Damage Biomarkers

The activity of plasma AST and ALT were estimated using a Transaminase CII-test kit (Wako, Japan).

### 4.6. CYP2E1 Activity Analysis

Livers were homogenized in nine volumes of tris HCl buffer (containing 0.25 M sucrose, pH 7.4) using a Polytron 1600E (Central Science Trade Co., Inc., Tokyo, Japan). The homogenates were centrifuged at 700× *g* for 10 min at 4 °C. The supernatant was collected as an S9 fraction. The activity of CYP2E1 was determined by the rate of hydroxylation of PNP measured at 546 nm [37]. The S9 fraction was added to 100 mM KH_2_PO_4_ (containing 0.2 mM PNP and 2.0 mM NADPH, pH 6.8) and incubated in a 37 °C water bath for 20 min. The reaction was stopped using 0.6 M perchloric acid (250 μL) and 10 M NaOH (75 μL) was added to the remaining supernatant. The results were expressed as the amount of *p*-nitrophenol pmols/min/mg protein formed and the determined concentration of 4-nitrocatechol (ε = 10. 28 mM^−1^·cm^−1^).

### 4.7. Measurement of Lipid Peroxidation

Lipid peroxidation was measured according to the method described by Ohkawa using a colorimetric reaction with thiobarbitric acid (TBA) and the measured lipid peroxidation was expressed as malondialdehyde (MDA) [38]. The frozen liver samples were excised and homogenized in nine volumes of ice-cold 1.15% KCl. Samples consisting of less than 0.2 mL of 10% (*w*/*v*) tissue homogenate were then added to 0.2 mL of 8.1% sodium dodecyl sulfate, 1.5 mL of 20% acetic acid solution adjusted to pH 3.5 with NaOH, and 1.5 mL of a 0.8% aqueous solution of TBA. The mixture was brought to a total volume of 4.0 mL using distilled water, mixed with 5.0 mL of a mixture of n-butanol and pyridine (15:1, *v*/*v*), and shaken vigorously. After centrifugation at 4000 rpm for 10 min, the organic layer was collected and its absorbance was measured at 532 nm. 1,1,3,3-Terramethoxypropane was used as an external standard. The level of lipid peroxidation was expressed in nmol of MDA.

### 4.8. Hepatocyte Preparation and Culture

Hepatocytes were isolated by collagenase perfusion following their removal from 10-week-old male Wistar rats anesthetized with sodium pentobarbital [39]. The viability of the isolated hepatocytes was greater than 90%, as determined by 0.2% trypan blue exclusion. The cells were plated on 35-mm plastic dishes at a density of 2.5 × 10^5^ cells/mL in 2 mL of Williams’ Medium E supplemented with 10% FBS. The cells were cultured in a humidified atmosphere (5% CO_2_/95% air) at 37 °C overnight. After pre-incubation, the cells were cultured in 10% FBS containing fresh Williams’ Medium E with different concentrations of ethanol, with or without DNSM, adenosine, or deoxyadenosine for 0–24 h.

### 4.9. Cell Viability Assay

The cell viability of the hepatocytes was measured by the Neutral Red assay, as previously described [40]. Neutral Red stock solution (0.4% Neutral Red in water) was diluted 1:80 in phosphate-buffered saline (PBS). Hepatocytes were incubated with the Neutral Red solution for 2 h at 37 °C to allow for the uptake of the lysosomal dye into viable cells. The Neutral Red solution was then removed, and the cultures were washed rapidly (in under 2.5 min) with a mixture of 1% formaldehyde-1% calcium chloride. A mixture of 1% acetic acid-50% ethanol was added to the cells at room temperature for 30 min to extract the Neutral Red from the hepatocytes. The optical density of each sample was then measured at 540 nm with a spectrophotometer. Cell viability was estimated as a percentage of the value obtained for untreated controls.

### 4.10. Assay of ADH Activities 

After incubation, the cells were washed twice and then dissolved with cold PBS. The debris was obtained by centrifugation at 2600× *g* for 1 min at 4 °C, and then buffer (50 mM HEPES pH 7.5, 0.25 M sucrose, 1 mM EDTA, 1 mM dithiothreitol (DTT), 3 mM MgCl_2_, 1 mM phenylmethylsulphonyl fluoride) was added. After two freeze-thaw cycles using liquid nitrogen, the cells were sonicated and centrifuged at 12,000× *g* for 20 min at 4 °C. Finally, the supernatant was collected. ADH activity was determined at 25 °C in a 1.5 mL volume (50 mM HEPES pH 8.0, 10 mM MgCl_2_, 1 mM DTT, 300 μM NAD^+^) in the presence or absence of ethanol (50 μL). The reaction was started by adding ethanol, and the absorbance at 340 nm was followed with a spectrophotometer. The linear initial increase in absorbance was used to determine specific enzyme activities with an absorption coefficient of 6.2 mM·cm^−1^.

### 4.11. Statistical Analysis

Statistical comparisons were performed between groups using one-way analysis of variance and post hoc multiple comparisons using Tukey’s test. A *p*-value less than 0.05 was considered significant.

## 5. Conclusions

In conclusion, we found that DNSM had protective effects against ethanol-induced liver injury. Although its precise mechanisms need to be elucidated further, DNSM may represent a novel tool for preventing alcohol-induced liver injury.

## Figures and Tables

**Table 1:** Changes in organ weight of rats.

Groups	Organ Weight (g)				
	Liver	Kidney	Spleen	Visceral Fat	Epididymal Fat
C	10.82 ± 0.38	1.60 ± 0.03	0.76 ± 0.04	6.52 ± 0.55	7.49 ± 0.61
ET	10.54 ± 0.46	1.58 ± 0.05	0.75 ± 0.03	4.54 ± 0.48	4.74 ± 0.37
T	12.35 ± 0.47	1.70 ± 0.08	0.72 ± 0.02	6.22 ± 0.52	6.02 ± 0.20
DNSM	10.76 ± 0.34	1.66 ± 0.02	0.70 ± 0.03	4.79 ± 0.52	5.44 ± 0.52

(C) Control diet; (ET) Control diet with 5% ethanol plus CCl_4_; (T) Control diet with CCl_4_; (DNSM) 0.12% DNSM diet with 5% ethanol plus CCl_4_.

**Table 2:** Composition of nucleotides and amino acids in DNSM.

Nucleotides *	Amount (g/100 g)
5′-dCMP	6.01
5′-dAMP	9.15
5′-dTMP	9.26
5′-dGMP	6.93
Total	31.35
**Amino Acids**	**Amount (g/100 g)**
Arg	17.80
Lys	2.66
His	0.65
Phe	0.89
Tyr	0.88
Leu	1.95
Ile	1.25
Met	0.60
Val	2.12
Ala	1.95
Gly	4.11
Pro	2.62
Glu	3.48
Ser	2.49
Thr	1.27
Asp	2.24
Trp	0.20
Cys	0.25
Total	47.41

* The amounts of nucleotides were analyzed after treatment of nuclease P_1_.

**Table 3:** Composition of diets.

Components (g)	Control	0.12% DNSM
Casein	200	200
l-Cystine	3	3
Cornstarch	397.486	396.286
α-Cornstarch	132	132
Sucrose	100	100
Soybean oil	70	70
Cellulose powder	50	50
Mineral mix (AIN-93G-MX) ^1^	35	35
Vitamin mix (AIN-93VX) ^2^	10	10
Choline hydrogen tartrate	2.5	2.5
t-Butylhydroquinone	0.014	0.014
DNSM	0	1.2
Total	1000	1000

^1^ Composition in g/kg diet: Calcium Carbonate, 357; Potassium Phosphate, Monobasic, 196; Potassium Citrate·H_2_O, 70.78; Sodium Chloride, 74; Potassium Sulfate, 46.6; Magnesium Oxide, 24; Ferric Citrate, 6.06; Zinc Carbonate, 1.65; Manganese Carbonate, 0.63; Cupric Carbonate, 0.324; Potassium Iodate, 0.01; Sodium Selenate, 0.01025; Chromium K Sulfate·12H_2_O, 0.275; Ammonium Molybdate·4H_2_O, 0.00795; Sodium Silicate·9H_2_O, 1.45; Lithium Chloride, 0.0174; Boric Acid, 0.0815; Sodium Fluoride, 0.0635; Nickel Carbonate·4H_2_O, 0.0306; Ammonium Vanadate, 0.0066; Sucrose, 221.0032; ^2^ Composition in g/kg diet: Vitamin A Acetate (500,000 IU/g), 0.8; Vitamin D3 (400,000 IU/g), 0.25; Vitamin E Acetate (500 IU/g), 15; Phylloquinone, 0.075; Biotin, 2; Cyanocobalamin, 2.5; Folic Acid, 0.2; Nicotinic Acid, 3; Calcium Pantothenate, 1.6; Pyridoxine-HCl, 0.7; Riboflavin, 0.6; Thiamin HCl, 0.6; Sucrose, 974.655.
